# Physical activity and nutrition intervention for Singaporean women aged 50 years and above: study protocol for a randomised controlled trial

**DOI:** 10.1186/s13063-018-2562-2

**Published:** 2018-04-27

**Authors:** Elaine Yee-Sing Wong, Andy H. Lee, Anthony P. James, Jonine Jancey

**Affiliations:** 1 School of Public Health, Curtin University, Perth, WA Australia; 2 Collaboration for Evidence, Research and Impact in Public Health, (CERIPH), School of Public Health, Curtin University, Perth, WA Australia

**Keywords:** Community - based, Healthy ageing, Intervention, Nutrition, Older women, Physical activity, Randomised controlled trial

## Abstract

**Background:**

The majority of the older Singaporean women aged 50 years and above are physically inactive and have unhealthy dietary habits, placing them at ‘high risk’ of non-communicable diseases (NCDs). The adoption of regular physical activity (PA) and a healthy diet are essential lifestyle behaviours to reduce this risk. This randomised controlled trial (RCT) involves the development, implementation and evaluation of a PA and nutrition programme for community-dwelling Singaporean women who currently attend recreational centres (RCs are public facilities supporting social leisure activities) in their local area. The intervention will be developed after conducting formative evaluation with RC attendees and managers through focus group discussions and pilot testing of resources (i.e. surveys, accelerometers, and health booklets). Programme ambassadors (trained, certified fitness instructors and nutritionists) will deliver all sessions in English and Mandarin; implement classes to meet participants’ varying needs; and conduct sessions at different times at convenient venues. Social Cognitive Theory (SCT) has been selected as the theoretical framework to inform intervention strategies as it explores the interactions of human behaviour with the environment and has been found to be valuable when developing behavioural change interventions particularly in older adults (J Gerontol B Psychol Sci Soc Sci 67B(1):18–26, 2012; Obesity Reviews 15(12):983–95, 2014). Its major construct, self-efficacy, is invaluable in achieving successful behaviour change, such as increasing levels of PA or improving dietary intake (Trials. 2017; 10.1186/s13063-016-1771-9; Psychol Health Med 18(6):714–24, 2013).

**Methods:**

The development and implementation of the PA and nutrition intervention strategies will be guided by SCT and Motivational interviewing (MI) and implemented by trained programme ambassadors at the RCs. Sixty RCs located in Singapore will be selected from five major geographical districts and randomly allocated to the intervention (*n* = 30) or control (*n* = 30) cluster. A sample of 600 (intervention *n* = 300; control *n* = 300) women aged 50 years and above will then be recruited from these 60 centres and only the intervention group will be enrolled into the PA and nutrition intervention. It is hypothesised that by the end of the intervention, the intervention group participants compared to the control group will show significantly greater improvements in the following outcome variables: PA and dietary behaviours, health-related quality of life, objective measures of PA, anthropometric, lipid and glucose profiles. Data will be collected at baseline and 6 months and analysed using mixed regression models.

**Discussion:**

It is anticipated that recruitment, retention and compliance of participants will be challenging due to the target group being unfamiliar with such community-based research programmes.

**Trial registration:**

Australian and New Zealand Clinical Trials Registry, ACTRN12617001022358. Registered on 14 July 2017. https://www.anzctr.org.au/Trial/Registration/TrialReview.aspx?id=372984&isReview=true

**Electronic supplementary material:**

The online version of this article (10.1186/s13063-018-2562-2) contains supplementary material, which is available to authorized users.

## Background

Singapore is a densely populated city with 5.6 million residents, comprising an ethnically diverse population of Chinese (74%), Malay (13%) and Indians (9%) [1], of which the proportion of dependent older adults is growing, representing a challenge for the health sector [2]. Compared to men, Singaporean women have a longer life expectancy [3]. They also have an increased risk of non-communicable diseases (NCDs), especially for those women aged 45 years and above [4]. Older women have been identified as a priority group to target in reducing the risk factors for all NCDs as the prevalence of high blood pressure (BP), cholesterol and diabetes (type 2) rise sharply for women from their 30s into their 60s [5]. In addition, compared to men, women living in Singapore aged 50 to 69 years have higher levels of abdominal fat (54% versus 9% in men), obesity (11% versus 8% in men), and lower levels of desirable highdensity lipoprotein-cholesterol (HDL-C), which is protective against atherosclerosis (5% versus 10% in men) [6]. Unhealthy lifestyle practices among these women, such as the high consumption of readily available low-nutrient, energy dense meals and physical inactivity [6, 7], contribute to their high rates of NCDs.

Adequate levels of physical activity (PA) and a nutritious diet have been recognised as crucial lifestyle factors for the prevention of NCDs [8]. However, based on the National Health Survey, Singaporean women aged over 50 years reported: (1) high levels of leisure time physical inactivity (72%), (2) low levels of regular exercise (16%) and (3) do not meet the recommended levels of PA for health benefit (39%) [6]. Reasons for low levels of participation include: a lack of time due to work and child commitments (47%); perception of doing adequate PA from undertaking housework (23%); and a lack of interest (16%) [6]. Results from the 2010 Singaporean National Nutrition Survey also revealed that a high proportion of Singaporean women aged 50–69 years have macronutrients intake exceeding the Recommended Dietary Allowance (RDA) for energy (58%), total fat (57%), saturated fat (67%), carbohydrate (54%) and protein (71%); but only 62% of them met the RDA for dietary fibre intake [7]. In addition, a lower percentage of these women met the recommended serving guidelines for vegetables (26%), fruits (34%), wholegrains (43%) and fare only slightly better for rice and alternatives (50%) as well as the meat and alternatives food groups (51%) [7].

Most Singaporeans (> 80%), including older adults, reside with their families in dense, affordable public housing [9]. Recreational centers (RCs) are built within this public housing to provide a venue for residents to come together with the intention of fostering community cohesiveness [10]. These RCs are in close proximity and easily accessed, providing an ideal setting to reach the target group and implement and evaluate a community-based PA and nutrition intervention for older women.

Despite the evidence that healthy lifestyle behaviours are linked to a reduction in NCDs [11], limited community-based intervention research has been undertaken in the over-50s target group in Asian communities [12]. The findings reported in the academic literature on the effectiveness of the PA and nutrition strategies in community-based interventions typically originate from Canada, US, Europe and Australia. For example, a systematic review of 17 community-based interventions indicated that PA and nutrition interventions incorporating face-to-face counselling and group session modes of delivery were most effective in a community-based setting [13]. Another review of nine randomised controlled trials (RCTs) reported that nutrition counselling involving active participation in health planning, goal setting, self-efficacy and collaboration were effective in influencing positive nutrition outcomes for older adults [14], while face-to-face meetings were found to be effective [15] due to the relationships that form during the exchange of information [16]. Significant improvement in dietary behaviour and PA levels has been observed in recent health interventions targeting older healthy adults in Asia using Social Cognitive Theory (SCT) [17-19]. Furthermore, a systematic review of SCT in interventions confirmed that it provided a practical and adequate theoretical framework for guiding early phases of intervention development for chronic health conditions [20]. Therefore, the proposed cluster RCT of SCT-based PA and nutrition intervention aims to develop and implement a culturally appropriate intervention to reduce the risk factors associated with NCDs for women aged 50 years and above who utilise RCs. It is hypothesised that levels of PA and dietary behaviours, lipid profiles and fasting blood glucose (BG) levels, as well as anthropometric measures, will demonstrate statistically significant improvements in the intervention group, when compared with the control group after the 6-month intervention.

## Methods

### Study design

**Fig. 1:**
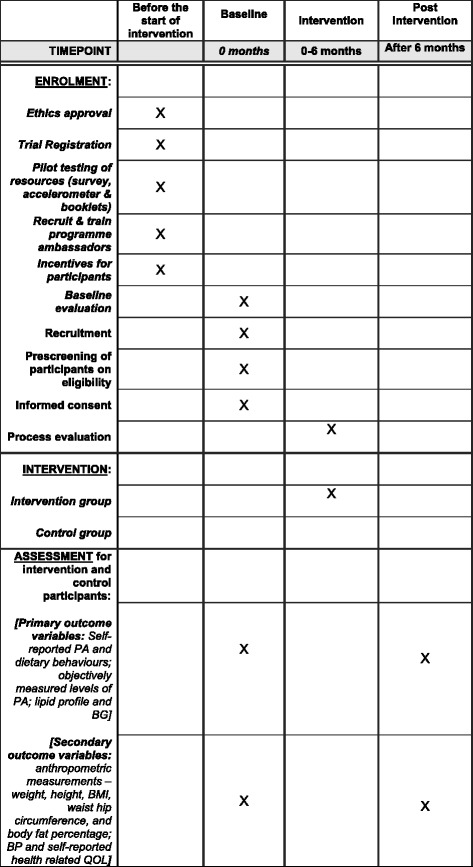
Standard Protocol Items: Recommendations for Interventional Trials (SPIRIT) Figure: proposed schedule for enrolment, intervention and assessment. *BG* blood glucose, *BMI* Body Mass Index, *BP* blood pressure, *PA* physical activity, *QOL* quality of life

**Fig. 2:**
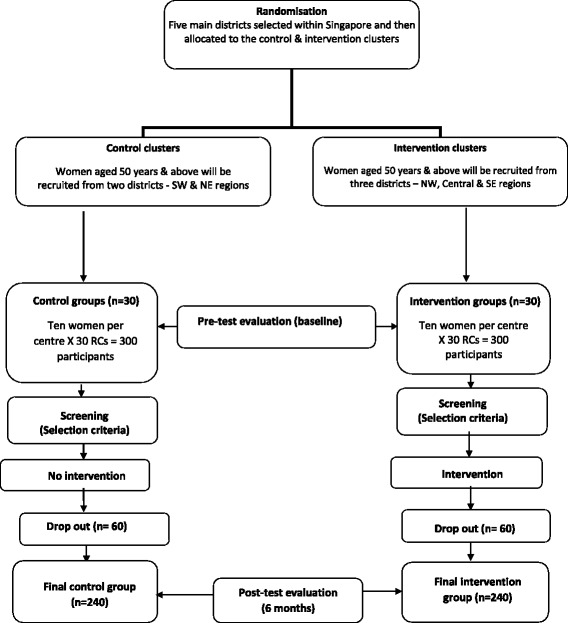
Flow chart of the research procedure. *NE* North East, *NW* North West, *RC* recreational centre, *SE* South East, *SW* South West

A 6-month, community-based PA and nutrition cluster RCT suited to the Singaporean context will be implemented and evaluated over two time points (baseline and 6 months); see Table 1. A proposed schedule for enrolment, intervention and assessment is shown in the Standard Protocol Items: Recommendations for Interventional Trials (SPIRIT) Figure (Fig. 1) and recommended items to address for intervention trials are reflected in the Additional file 1: SPIRIT Checklist [21]. A flow chart of the research procedure is displayed in Fig. 2.

**Table 1:** Proposed pre-post data collection and intervention

**Study group**	**(Baseline) 0 month**	**Intervention**	**(Post intervention) 6 months**
Controls (*n* = 300)	O1		O2
Interventions (*n* = 300)	O1	X	O2

*O* observation, *X* intervention

### Study procedure

#### Recruitment of RCs

Singapore is divided into five geographical districts, as seen in Additional file 2 [22, 23], which are all similar in terms of socioeconomic status [24]. To enhance the chance of recruiting participants into the intervention programme, three out of the five districts were randomly allocated to the intervention cluster (by drawing names from a bag) and the remaining two districts to the control cluster. The process resulted in the intervention sites (districts: 2-North West, 3-Central, and 5-South East) and control sites (districts: 1-South West, and 4-North East). Sixty RCs (intervention *n* = 30, control *n* = 30) will be randomly selected from these geographical districts. Due to the differences in the number of RCs within the districts, approximately 9–12% of the listed RCs in each district will be chosen using computer-generated random numbers. These RCs will have a minimum separation distance of 4 km to avoid risk of contamination between intervention and control groups. The recruitment process will continue until 30 control and 30 intervention RCs consent to take part in the study. The participants will all be blinded to their group status (intervention and control) during the recruitment and intervention period.

#### Recruitment of participants

A sample of 600 (interventions *n* = 300; controls *n* = 300) women will then be recruited from these 60 RCs. Participants are required to be: (1) female, aged 50 years and above, (2) undertaking less than 150 min of moderate intensity leisure PA per week (self-reported), (3) not have any medical condition that prohibits involvement in a PA programme, and (4) not currently enrolled in other nutrition and PA research studies.

#### Procedure

Programme recruitment flyers will be placed on the RC’s bulletin boards and promoted by the RC managers. Those residents indicating to the RC managers that they are interested in the programme and agreeing to provide their contact details will have their telephone number provided to the research team. These participants will then be telephoned to explain the purpose of the study and to determine their eligibility based on the selection criteria. After the initial telephone screening, eligible participants will be invited to take part, informed of their rights and confidentiality issues, and that they are free to withdraw at any stage.

At the initial meeting, baseline data will be collected and recorded using the health surveys available in the English and Chinese language reflected in the Additional files 3 and 4 [25-27]. Trained researchers (final-year tertiary students) will collect self-reported data on dietary habits and PA levels used to assess current dietary and PA levels and undertake anthropometric measures. These programme ambassadors are final-year nutrition, sports and wellness undergraduate students who will conduct their activities under close supervision of qualified nutritionists and certified fitness instructors. The principal investigator (PI) will fit and explain the management of the accelerometers. Fasting blood samples will be collected by a phlebotomist. Any participants exhibiting hypercholesterolaemia (total cholesterol (TC) ≥ 6.2 mmol/L) [28] or hyperglycaemia (BG > 7 mmol/L) levels [29] will be excluded from the study and referred to their medical practitioner for follow-up. Participants on medication will not be excluded and both the pre and post survey will document all those who are on medication including the type of medication and subgroup analysis will be undertaken.

Both the intervention and control participants who undergo pre and post data collection will be given an incentive of free health products (health calendar, sports towel, writing pad and pen with health messages) and supermarket vouchers in appreciation of their involvement in the study. However, the control group will be blinded to the nature of the intervention and instead only receive a falls prevention booklet.

#### Sample size determination

The power calculations are based on a logistic mixed regression model with the prevalence of moderate intensity PA participation as the outcome variable. An appropriate sample size for this study is determined in a similar manner to previous studies which examined effective recruitment and retention of older adults in a PA programme [30]; PA and nutrition intervention for seniors [31]; as well as PA and nutrition intervention targeting middle-aged adults [32]. For the mixed regression analyses when adjusting for the clustering of RCs, a sample size of *n* = 480 participants (240 participants per group) will allow for an 80% statistical power to detect a medium effect size of 10% improvement in PA prevalence (sufficient leisure time PA estimated to be 28% at baseline) [6] by the intervention group relative to the control group at a significance level of 5%. To account for a 20% attrition rate between the two time points (pre and post intervention), a total of *n* = 600 adult women will be initially recruited at baseline. The sample size takes non-respondents into consideration within the duration of the study but without covariate adjustment.

### Outcome measures

Table 2 summarises the outcome variables and how they relate to their corresponding measurement tools.

**Table 2:** Primary and secondary outcome variables and measuring tools

**Primary outcome variables**	**Measurement tools**
Self-reported levels of PA behaviour (incidental, programmed and sedentary)	GPAQ
Objectively measured levels of PA: intensity, duration and frequency	ActiGraph GT3X accelerometers
Self-reported levels of dietary behaviours on wholegrains, fat, oils, salt, sugar, fruits and vegetables consumption	STEPS dietary behaviour questionnaire
Lipid profile – cholesterol and TG, BG	Fasting blood samples
**Secondary outcome variables**	**Measurement tools**
Anthropometric measurements – weight, height, BMI, waist hip circumference and body fat percentage	Portable stadiometer, calibrated weighing machines, tape measures and Endo Body Fat Composition Analyser E-DBS908
BP	Omron electronic BP monitor
Self-reported health-related QOL	SF-8 questionnaire

*BG* blood glucose, *BMI* Body Mass Index, *BP* blood pressure, *GPAQ* Global Physical Activity Questionnaire, *PA* physical activity, *QOL* quality of life, *SF* Short Form, *TG* triglyceride

#### Primary outcome variables

##### Self-reported PA behaviour

will be obtained through the administration of the Global Physical Activity Questionnaire (GPAQ), a widely used instrument specifically developed by the World Health Organisation (WHO) for population-wide PA surveillance, and is available in the Chinese language [33]. This instrument has acceptable measurement properties and was used in the National Health Survey to collect data on Singaporeans aged 18–69 years on PA levels [6]. In Singapore, this tool has demonstrated fair-to-moderate correlations for moderate-to-vigorous PA for validity and moderate levels of reliability [34].

##### Accelerometers

(ActiGraph GT3X) [35] will be explained and fitted to the intervention participants. They will wear this device on their right hip for seven consecutive days at baseline and 6 months (post intervention) and remove it when sleeping, showering or doing water-based activities. Data collected will be downloaded using the ActiLife 6 software. This information will be summarised into daily average counts (counts per min) and duration of activity (min per day) at specific intensity levels (inactive, light, moderate, and vigorous) [36].

##### Self-reported dietary eating habits

will be assessed through a WHO-developed, standardized STEPS dietary behaviour questionnaire to monitor the main NCD risk factors [37]. This is a feasible, low-cost nutrition assessment tool comprising 21 questions measuring dietary consumption of wholegrains, fat, oils, salt, sugar, fruits and vegetables for the past week [26].

#### Blood samples

A fasting (> 10 h) blood sample will be collected from the antecubital vein of all participants by trained phlebotomists into appropriate vacuum tubes [38]. All blood analysis will be carried out at the Quest Lab pathology laboratory using an Abbott Architect auto-analyser according to the manufacturer’s instructions. The assays for TC, triglyceride (TG) and glucose are all based on standard enzymatic colourimetric methodology [39, 40]. Serum low-density lipoprotein-cholesterol (LDL-C) will be calculated by the modified Friedewald equation and non-HDL-C will be calculated by subtracting the HDL-C from the TC [41, 42].

#### Secondary outcome variables

##### Weight

will be measured (wearing light clothing without shoes) using a calibrated electronic scale and recorded to the nearest 0.01 kg while *height* will be measured with a portable stadiometer to the nearest 0.1 cm (barefooted) [43, 44].

##### Body Mass Index

(kg/m^2^) will be calculated as weight in kilograms divided by the square of height in metres [45].

##### Body fat percentage

will be measured by an Endo Body Fat Composition Analyser E-DBS908 (without shoes and socks) [46].

##### Waist circumference

will be measured by a non-stretch tape, standing up at the level midway between the lowest rib margin and the iliac crest and recorded to the nearest 0.1 cm [47].

##### Hip circumference

will be measured at the widest circumference at the level of the symphysis pubis and gluteus maximus [32].

##### Waist hip ratio

will be calculated as waist circumference divided by hip circumference [48].

##### Systolic and diastolic BP

will be taken from the left arm after consecutive measurements of the participants seated down and rested with an appropriate-sized cuff placed at the heart level using an Omron electronic BP monitor [49, 50].

##### Self-reported health-related quality of life (QOL)

using the Short Form (SF)-8 questionnaire will assess the health-related QOL and provide a brief overview of the physical, psychological and social domains of health to further support the study [51, 52].

##### Demographic data

including age, ethnicity, education, marital status, existing medical conditions, type of medications use and type of housing dwelling will be collected.

### Process evaluation

This will be undertaken throughout the intervention programme to determine reach (attendance); fidelity (quality of programme, programme delivery and resources), together with recruitment, dose delivered and received [53]. Criteria for assessing programme quality will take into account: (1) utility: information needs of participants are satisfied, e.g. usefulness of resources; (2) feasibility: cost-effectiveness, e.g. efficiency of evaluation process to justify use of available resources; (3) propriety: ethical standards are met, e.g. aggregated evaluation findings are made accessible to participants; and (4) accuracy: valid and reliable information, e.g. clear and accurate report documentation [54]. Structured feedback forms and focus group interviews will be undertaken with the participants to determine the effectiveness of programme and programme ambassadors; explore barriers, motivators; and satisfaction levels [55]. Collection of these data will provide information on the effectiveness of strategies, applicability of resources, and other factors that impact upon the delivery of the intervention [56]. These pertinent details will provide insight into programme implementation and factors that influence participation.

Exit interviews will be undertaken with programme completers (*n* = 12) and non-completers (*n* = 12). This sample size is considered adequate for non-probabilistic purposeful sampling [57]. Informed verbal consent will be obtained prior to conducting the interviews. The interviews will explore perception of the resources, the intervention and the programme ambassadors. Reasons for programme withdrawal will be obtained from non-completers. It is estimated that the face-to-face exit interviews will be of 20-min duration.

### Theoretical framework

Theoretical perspective underpinning this study is the SCT, which identifies the interaction of the individual with the social and physical environment, and how this interaction influences health behaviours in older adults [58-60]. It specifies a set of psychosocial constructs that include self-efficacy; outcome expectations (cognitive influences); observational learning and social support (environmental influences); as well as goal-setting and reinforcement (behavioural influences) which are described in Table 3 [61, 62]. In addition, Motivational Interviewing (MI) techniques will be used during the intervention delivered by the programme ambassadors. This is a person-centred, collaborative communication style, to raise commitment and reinforce behaviour change [63]. Studies on applied MI strategies have demonstrated its practicality in positively tackling unhealthy dietary and low PA levels [64, 65]. The use of these techniques in this study will support autonomy and heighten intrinsic motivation towards healthier lifestyles.

**Table 3:** Application of Social Cognitive Theory (SCT) to inform strategies and methods

**SCT**	**Strategies**	**Methods**
Environment	Build physical and nutrition supports within the RCs	Conduct face-to-face meetings, follow-up calls and provide educational resources
Outcome expectations / Expectancies	Educate on the benefits of nutritious diet and regular PA / achievement of better health screening results and MI	Introduce concept of goal setting and resources at initial meeting; undertake face-to-face sessions, follow-up calls and feedback sessions
Self-efficacy	Goal setting, monitor progress and mastery of health practices and MI	Regular coaching and feedback on participants' PA and nutrition goals towards improving health outcomes (incremental and achievable). Programme ambassadors encourage the adoption of health-enhancing behaviour and practice of new skills and provide feedback
Observational learning	Observe programme ambassadors’ dietary and PA behaviours	Demonstrate PA and showcase cooking advice practices. Programme ambassadors act as positive role models
Positive reinforcement	Education and skill building sessions and MI	Regular encouragement, ongoing monitoring of personal PA goals through follow-up support and feedback

*MI* motivational interviewing, *PA* physical activity, *RC* recreational centre, *SCT* social cognitive theory

### Intervention

This 6-month community-based Singapore Physical Activity and Nutrition Study (SPANS) intervention programme will be based on the Singaporean PA and nutrition guidelines [66-68]. Programme ambassadors will undergo comprehensive training in MI and intervention programme and will undertake all the intervention activities and distribute the resources bi-weekly (see Table 4).

**Table 4:** Singapore Physical Activity and Nutrition Study (SPANS) intervention

**Session (timing)**	**Session details**	**Participant’s resources / interactive activities**
**PA sessions** **(1 hr- twice a month)** **[Week 2 -24]**	1. Introduction to SPANS programme 2. **Focus PA:** aerobic • Benefits/barriers/motivators • Guidelines 3. Establish short and long term PA goals	1. **Resources** • ‘Active for Life’ booklet 2. **Interactive activities** • PA warm up • Muscle strengthening and low intensity aerobic workout • Cool down and stretches
**Nutrition session 1 – Eat Well, Live Well** **[Week 1]**	1. **Focus on nutrition** • Benefits/barriers/motivators • Healthy plate model and guidelines 2. Establish short and long term nutrition goals	1. **Resources:** • ‘Nutrition Guide for Healthy Aging’ booklet • Health calendar with monthly recipes and health tips 2. **Interactive activities:** • What’s On Your Plate? • Read your food label
**Nutrition session 2 – Smart Shopping and Dining Alert [Week 12]**	1. **Focus on nutrition** • Healthy shopping and cooking tips • Healthier Choice Symbols	1. **Resources** • ‘Recipes for Health Aging’ booklet • ‘Lowering BP’ booklet 2. **Interactive activities** • Ranking game on food calorie • Guess the herbs and spices in cooking
**Nutrition session 3 – Take Charge Of Your Health** **[Week 24]**	1. **Focus on nutrition and PA** • Manage cholesterol and BG levels 2. Identify social support and overcome relapse	1. **Resources** • ‘Keeping Cholesterol in Check’ booklet • ‘Tips to Better Health: Keeping My Blood Sugar Levels Healthy’ booklet 2. **Interactive activities** • Mix and match cholesterol in foods • Brainstorm on improving BG levels
**PA and nutrition counselling** **(45 mins – once every 2 months)** **[Week 4, 12, 20]**	1. **Focus on behavioural change** 2. Record dietary and PA record 3. Set, review short and long term goals 4. Provide guidance and support on healthy lifestyle	1. **Resources** • Fridge magnet with reminders on ‘Eat More Fruits and Vegetables’ • Tote Bag on ‘Healthy Living’ • Sports towel with ‘Warm Up Before PA’ slogan 2. **Interactive activity** • Goal setting and reflection
**Follow-Up Calls / PA and nutrition text phone messages** **(once per month)** **[Week 4 -24]**	1. A monthly PA and nutrition ‘tip of the day’ will be sent via a “WhatsApp” chat platform.	**Interactive activity** • Participants send other health-related messages to simulate discussion.

#### Group PA, nutrition talks, dietary counselling sessions and telephone contact

Bi-weekly, low-intensity PA, strength and balanced exercises sessions will be conducted. There will be bimonthly nutrition talks, bimonthly dietary counselling and monthly follow-up calls incorporating MI. Programme ambassadors will follow-up with participants’ enquires and provide feedback to support the adoption of healthy practices and monitor the progress of their participants’ goals. Participants will be encouraged to do moderate intensity PA for at least 150 min per week and reminded to monitor their daily progress and goals by recording the frequency and duration of PA in a health calendar provided at the commencement of the programme. Goal setting and feedback will be emphasized at all contact points during the intervention.

#### Educational resources

The programme ambassadors will introduce the 6-month intervention; distribute the resources throughout the intervention period; and ask participants to use the booklets as a guide to healthy ageing.

##### *Booklets*

1. ‘*Active for Life*’: benefits of PA; guidelines for frequency, intensity, time and type of PA; and safety issues
2. ‘*Recipes for Healthy Ageing*’*– Nutrition Guide and Recipes*: my healthy plate model; dietary guidelines for older adults; tasty recipes; and healthy eating tips
3. ‘*Lowering BP*’ booklet: healthy BP range; problems of high BP levels; dietary sources of sodium; and ways to control and manage BP levels
4. ‘*Keeping Cholesterol in Check*’: dietary sources of cholesterol; ways to reduce dietary cholesterol; and recipe modification for better heart health
5. ‘*Tips to Better Health – Keeping my Blood Sugar Levels Healthy*’: pre-diabetes and diabetes; issues of high BG levels; and how do I control my BG levels?

#### Calendar

Participants will receive easy, healthy recipes found to enhance the adoption of healthy dietary behaviours among women of lower health literacy levels [69] in a *‘health calendar’* that contains health tips acting as reminders to stay active and eat healthily.

(These resources are available from the first author upon request).

#### Health text messaging

A nutrition and PA ‘tip of the month’ will be sent to all participants as text messages via "WhatsApp" on a monthly basis to motivate them to practice healthy lifestyle habits.

### Statistical analysis

After data collection, outcome variables of interest, such as blood profiles, self-reported PA and dietary behaviours, and anthropometric measures will be examined and compared between subgroups of interest via the Statistical Package for the Social Science version 24 [70]. Descriptive and summary statistics will be used to quantify participants’ characteristics and outcome variables. Non-parametric statistics will be applied whenever non-normality of the outcomes is detected. Multi-variable mixed regression analyses will be used to confirm the effects of the proposed intervention, taking into account the repeated measures (at two time points) and the clustering of the observations. All variables will be entered using the stepwise regression. The regression model will also account for effects of potential confounders (age, ethnicity, education, marital status, consumption of medication and housing type).

All qualitative data from process evaluation will be transcribed, with at least 10% of all transcribed data randomly selected and reviewed for each transcription. Transcribed data will be coded to create common themes or categories. Data will be collated, presented thematically and supported by direct quotes from participants. Data management of full transcripts and other texts will be facilitated by the NVivo software package [71] to conduct a framework analysis [72, 73]. Participants will not be identified in any transcription.

## Discussion

Given the less than optimal dietary habits and low levels of PA among Singaporean women, it is imperative to develop appropriate lifestyle interventions to support the adoption of health-enhancing lifestyle behaviours. RCs provide a community setting to reach this target group to implement a programme that supports an increase in knowledge and development of skills to encourage behaviour change. The proposed study represents the first PA and nutrition cluster RCT located in RCs to be conducted in Singapore with older women. It will give valuable information on PA and nutrition behaviours to enhance healthy ageing outcomes. Moreover, findings from this study may provide insights and recommendations for policy-makers and key stakeholders to develop, modify or create new healthy living RCs with supportive environments in the future.

## Trial status

Recruitment of intervention and control group participants, was still ongoing at the time of manuscript submission.

## Additional files

Additional file 1: SPIRIT 2013 Checklist: recommended items to address in a clinical trial protocol and related documents. (DOC 127 kb)

Additional file 2: Stratified cluster random sampling of the 5 major districts in Singapore for the physical activity (PA) and nutrition intervention. (DOC 169 kb)

Additional file 3: Health survey: English. (PDF 318 kb)

Additional file 4: Health survey: Mandarin. (PDF 553 kb)

## Abbreviations

BG: Blood glucose
BMI: Body Mass Index
BP: Blood pressure
GRC: Group representative constituency
GPAQ: Global Physical Activity Questionnaire
HDL-C: High-density lipoprotein-cholesterol
LDL-C: Low-density lipoprotein-cholesterol
MI: Motivational Interviewing
NCDs: Non-communicable diseases
PA: Physical activity
PI: Principal investigator
QOL: Quality of life
RC: Recreational centre
RCT: Randomised controlled trial
RDA: Recommended Dietary Allowance
SCT: Social Cognitive Theory
SF: Short Form
SMC: Single member constituency
SPANS: Singapore Physical Activity and Nutrition Study
SPIRIT: Standard Protocol Items: Recommendations for Interventional Trials
TC: Total cholesterol
TG: Triglyceride
WHO: World Health Organisation
