# Supplementary material for: Physical activity and nutrition intervention for Singaporean women aged 50 years and above: study protocol for a randomised controlled trial
Source: Trials. 2018 Apr 27;19:257. doi: 10.1186/s13063-018-2562-2 (PMC5923190; doi:10.1186/s13063-018-2562-2)
Supplement: Supplementary file 2 — Stratified cluster random sampling of the 5 major districts in Singapore for the physical activity (PA) and nutrition intervention. (DOC 169 kb) [file 13063_2018_2562_MOESM2_ESM.doc]

**FIG 3:**

**Stratified cluster random sampling of the 5 major districts in Singapore for the PA and Nutrition Intervention**

District 2 (n=106) Sample intervention clusters = 10 (9%)


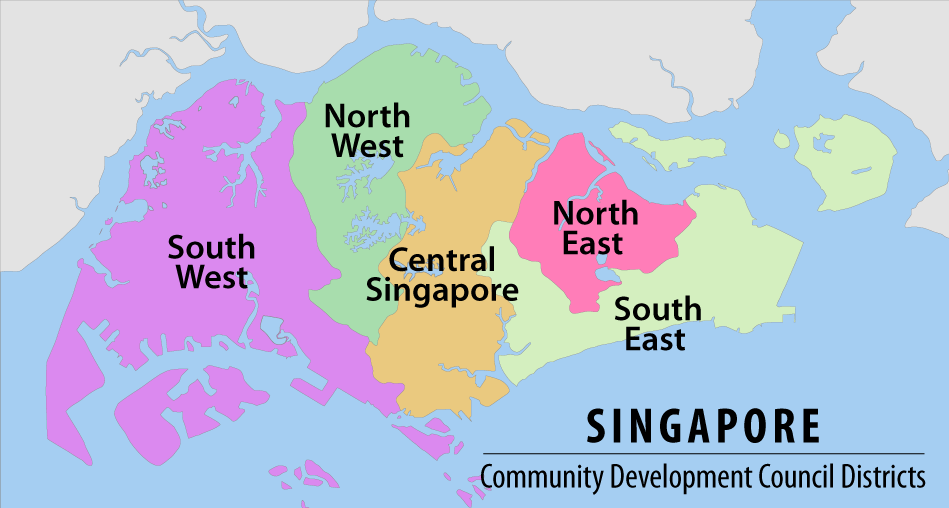


District 1 (n=115) Sample control clusters =14 (12%)

District 3 (n=164) Sample intervention clusters = 14 (9%)

District 4 (n=140) Sample control clusters = 16 (11%)

District 5 (n= 54) Sample intervention clusters = 6 (11%)

**Legend:**

District 1 – South West (Control RC clusters)

District 2 – North West (Intervention RC clusters)

District 3 – Central Singapore (Intervention RC clusters)

District 4 – North East (Control RC clusters)

District 5 – South East (Intervention RC clusters)

**Note:** All of the 5 major districts vary in the number of residential centres (RCs). Thus, a 9-12% of a subsample of RCs will be randomly stratified from each major districts to determine intervention cluster RCs (n=30) and the control cluster RCs (n=30) for the PA and nutrition intervention.

**The South West district covers 17 areas (District 1):**

- Chua Chu Kang GRC comprising of Bukit Gombak, Chua Chu Kang, Keat Hong, Nanyang
- Jurong GRC comprising Jurong Central, Jurong Spring, Taman Jurong, Clementi, Bukit Batok East
- West Coast GRC comprising of Ayer Rajah, Boon Lay, Telok Blangah and West Coast
- SMCs of Bukit Batok, Hong Kah North, Pioneer and Yuhua

**The North West district covers 19 areas (District 2):**

- Holland-Bukit Timah GRC comprising Bukit Timah, Cashew, Ulu Pandan and Zhenghua
- Sembawang GRC comprising Admiralty, Canberra, Gambas, Sembawang and Woodlands
- Nee Soon GRC comprising Chong Pang, Kebun Baru, Nee Soon Central, Nee Soon East and Nee Soon South
- Marsiling-Yew Tee GRC comprising Limbang, Marsiling, Woodgrove, Yew Tee
- SMC of Bukit Panjang

**The Central Singapore district covers 23 areas (District 3):**

- Ang Mo Kio Group Representative Constituency (GRC) comprising Ang Mo Kio-Hougang, Cheng San-Seletar, Jalan Kayu, Sengkang South, Teck Ghee and Yio Chu Kang
- Bishan-Toa Payoh GRC comprising Bishan East- Thomson, Bishan North, Toa Payoh West- Balestier, Toa Payoh Central and Toa Payoh East - Novena
- Jalan Besar GRC comprising Kampong Glam, Kolam Ayer, Kreta Ayer-Kim Seng and Whampoa
- Tanjong Pagar GRC comprising Buona Vista, Moulmein-Cairnhill, Queenstown, Henderson-Dawson and Tanjong Pagar-Tiong Bahru
- Single Member Constituency (SMC) of Radin Mas, Sengkang West and Potong Pasir

**The North East district covers 18 areas (District 4):**

- Aljunied GRC comprising Bedok Reservoir-Punggol, Eunos, Kaki Bukit, Paya Lebar and Serangoon
- Pasir Ris-Punggol GRC comprising Pasir Ris East, Pasir Ris West, Sengkang Central, Punggol North, Punggol Coast and Punggol West
- Tampines GRC comprising Tampines Central, Tampines Changkat, Tampines East, Tampines North and Tampines West
- SMCs of Hougang and Punggol East

**The South East district covers 12 areas (District 5):**

- East Coast GRC comprising Bedok, Changi-Simei, Siglap, Kampong Chai Chee
- Marine Parade GRC comprising Braddell Heights, Geylang Serai, Kembangan-Chai Chee, Joo Chiat, Marine Parade
- SMCs of Fengshan, Macpherson and Mountbatten
