# Supplementary material for: Physical activity and nutrition intervention for Singaporean women aged 50 years and above: study protocol for a randomised controlled trial
Source: Trials. 2018 Apr 27;19:257. doi: 10.1186/s13063-018-2562-2 (PMC5923190; doi:10.1186/s13063-018-2562-2)
Supplement: Supplementary file 3 — Health survey: English. (PDF 318 kb) [file 13063_2018_2562_MOESM3_ESM.pdf]

For Office Use

HEALTH SURVEY

PERSONAL DETAILS

Code
Q1 [ ]
Q2 [ ] ____________
Q3 [ ]
Q4 [ ]
Q5 [ ] ____________
Q6 [ ] ____________

1. What is your age?

________ years

For questions 2 & 3 please only cross out only one box.

2. What is your ethnicity?

Chinese………………………… ☐1 Malay…………………………... ☐2

Indian …………………………… ☐3 Other________________________ ☐9

3. Which of the following best describes the highest level of education you have completed?

Primary school or no education …… ☐1 High/secondary school ………… ☐2

Diploma …………………………… ☐3 University/tertiary education & above …… ☐4

4. Marital Status.

Married…..... ☐1 Widowed ……… ☐2 Single ……… ☐3 Divorced ……… ☐4

5. Do you have any of the following? (cross out all that apply)

Heart disease …… ☐1 Stroke ……… ☐2 High blood pressure …..... ☐3

Diabetes ………… ☐4 Cancer ……... ☐5 High cholesterol …..…… ☐6

Osteoporosis ……. ☐7 Arthritis ……. ☐8 Other: __________________ ☐9

6. Are you taking any of the following medications? (cross out all that apply)

Blood pressure medication (e.g. Ramipril)……………………………………………. ☐1

Cholesterol medication (e.g. Lipitor) …………………………………………………. ☐2

Blood clotting/Anti-platelet medication (e.g. Aspirin) ………………………………….. ☐3

Beta-blockers (e.g. Cartrol/Cartia) ……………………………………………………. ☐4

Others: ____ ☐9

**7. Type of housing are you residing in.**<br>2 Room HDB Flat ………………………………………………………… [ ] 1<br>3 Room HDB Flat ………………………………………………………… [ ] 2<br>4 Room HDB Flat ………………………………………………………… [ ] 3<br>5 Room HDB Flat ………………………………………………………… [ ] 4<br>Others: ______________________________ [ ] 9	Q7 [ ]<br>__________

Physical Activity

Next I am going to ask you about the time you spend doing different types of physical activity in a typical week. Please answer these questions even if you do not consider yourself to be a physically active person.<br>Think first about the time you spend doing work. Think of work as the things that you have to do such as paid or unpaid work, household chores i.e. vacuuming, mopping, scrubbing floors and shopping for groceries etc. In answering the following questions 'vigorous-intensity activities' are activities that require hard physical effort and cause large increases in breathing or heart rate, 'moderate-intensity activities' are activities that require moderate physical effort and cause small increases in breathing or heart rate.	**Code**
8. **Does your work involve vigorous-intensity activity that causes large increases in breathing or heart rate like carrying or lifting heavy loads, mopping or scrubbing floors for at least 10 minutes continuously?**<br>Yes …………………… [ ] 1<br>[ ] 2 No → ***Skip to question 11***	Q8 [ ]
9. **In a typical week, on how many days do you do vigorous-intensity activities as part of your work?**<br>_____ days per week	Q9 [ ]
10. **How much time do you spend doing vigorous-intensity activities at work on a typical day?**<br>_____ hours per day<br>_____ minutes per day	Q10 [ ]
11. **Does your work involve moderate-intensity activity that causes small increases in breathing or heart rate such as brisk walking, carrying light groceries, sweeping, washing windows for at least 10 minutes continuously?**<br>Yes …………………... [ ] 1<br>[ ] 2 No → ***Skip to question 14***	Q11 [ ]

12. **In a typical week, on how many days do you do moderate-intensity activities as part of your work?**<br><br>_____ days per week	Q12 [ ]
13. **How much time do you spend doing moderate-intensity activities at work on a typical day?**<br><br>_____ hours per day<br><br>_____ minutes per day	Q13 [ ]
The next questions exclude the physical activities at work that you have already mentioned. Now I would like to ask you about the usual way you travel to and from places. For example to work, for shopping, to market, to place of worship.	
14. **Do you walk or use a bicycle (*pedal cycle*) for at least 10 minutes continuously to get to and from places?**<br><br>Yes ………………….... ☐ 1<br><br>☐ 2 No → ***Skip to question 17***	Q14 [ ]
15. **In a typical week, on how many days do you walk or bicycle for at least 10 minutes continuously to get to and from places?**<br><br>_____ days per week	Q15 [ ]
16. **How much time do you spend walking or bicycling for travel on a typical day?**<br><br>_____ hours per day<br><br>______ minutes per day	Q16 [ ]
The next questions exclude the work and transport activities that you have already mentioned. Now I would like to ask you about sports, fitness and recreational activities (leisure).	
17. **Do you do any vigorous-intensity sports, fitness or recreational (leisure) activities that cause large increases in breathing or heart rate like running, swimming or dancing such as quick step or salsa, aerobics, playing ball sports i.e. soccer, volleyball, basketball, netball or badminton for at least 10 minutes continuously?**<br><br>**Yes** ………………….... ☐ 1<br><br>☐ 2 **No → Skip to question 20**	Q17 [ ]

18. **In a typical week, on how many days do you do vigorous-intensity sports, fitness or recreational (*leisure*) activities?**<br><br>_____ **days per week**	Q18 [ ]
19. **How much time do you spend doing vigorous-intensity sports, fitness or recreational activities on a typical day?**<br><br>_____ **hours per day**<br><br>_____ **minutes per day**	Q19 [ ]
20. **Do you do any moderate-intensity sports, fitness or recreational (leisure) activities that causes a small increase in breathing or heart rate such as brisk walking, gardening, doing low-aerobic exercise, using hand weights, using resistance band, doing Qigong or Tai chi for at least 10 minutes continuously?**<br><br>Yes ………….……..... ☐ 1<br><br>☐ 2 No → ***Skip to question 23***	Q20 [ ]
21. **In a typical week, on how many days do you do moderate-intensity sports, fitness or recreational (*leisure*) activities?**<br><br>_____ **days per week**	Q21 [ ]
22. **How much time do you spend doing moderate-intensity sports, fitness or recreational (*leisure*) activities on a typical day?**<br><br>_____ **hours per day**<br><br>_____ **minutes per day**	Q22 [ ]
The following question is about sitting at work, lying on sofa at home, getting to and from places, or with friends including time spent sitting at a desk, sitting with friends, travelling in car, bus, train, reading or watching television, but do not include time spent sleeping.	
23. **How much time do you usually spend sitting or lying on a sofa on a typical day?**<br><br>_____ **hours per day**<br><br>_____ **minutes per day**	Q23 [ ]

Nutrition

The next questions ask about the fruits and vegetables that you usually eat. I have a nutrition poster here that shows you some examples of local fruits and vegetables. Each picture represents the size of a serving. As you answer these questions please think of a typical week in the last year.	Code
24. **In a typical week, on how many days do you eat fruit?**<br>_____ **days per week**<br>If Zero days, go to Q26.	Q24 [ ]
25. **How many servings of fruit do you eat on one of those days?**<br>______ **of servings**	Q25 [ ]
26. **In a typical week, on how many days do you eat vegetables?**<br>_____ **days per week**<br>If Zero days, go to Q28.	Q26 [ ]
27. **How many servings of vegetables do you eat on one of those days?**<br>______ **of servings**	Q27 [ ]
With the next questions, we would like to learn more about salt in your diet. Dietary salt includes ordinary table salt, unrefined salt such as sea salt, salty stock cubes and powders, and salty sauces such as soya sauce or fish sauce. The following questions are on adding salt to the food right before you eat it, on how food is prepared in your home, on eating processed foods that are high in salt such as luncheon meat, salted fish, Szechuan vegetable, preserved radish, preserved cai xin, salted egg, chinese sausage (lap cheong), fermented beancurd, and questions on controlling your salt intake. Please answer the questions even if you consider yourself to eat a diet low in salt.	
28. **How often do you add salt or a salty sauce such as soya sauce to your food right before you eat it or as you are eating it?**<br>Always (6-7 times per week) ……… ☐ 1 Rarely (once per month)… ☐ 4<br>Often (2-5 times per week) ……….. ☐ 2 Never ………….. ☐ 5<br>Sometimes (2-4 [1 time per week] times per month) … ☐ 3	Q28 [ ]

29. **How often is salt, salty seasoning or a salty sauce added in cooking or preparing foods in your household?**<br>Always (6-7 times per week) ……… 1 Rarely (once per month)… 4<br>Often (2-5 times per week) ……….. 2 Never ………….. 5<br>Sometimes (2-4 [1 time per week] times per month) … 3	Q29 [ ]
30. **How often do you eat processed food high in salt? By processed food high in salt, I mean foods that have been altered from their natural state, such as packaged salty snacks, canned salty food including pickles, Szechuan vegetables, and salty food prepared at a fast food restaurant, processed meat.**<br>Always (6-7 times per week) ……… 1 Rarely (once per month)… 4<br>Often (2-5 times per week) ……….. 2 Never ………….. 5<br>Sometimes (2-4 [1 time per week] times per month) … 3	Q30 [ ]
31. **How much salt or salty sauce do you think you consume?**<br>Far too much (7 times per week)…. 1 Sometimes(1-3 times per week] 3<br>Too much (4-6 times per week)……. 2 Rarely (once per month)..... 4	Q31 [ ]
32. **How important to you is lowering the salt in your diet?**<br>Very important ………. 1 Not at all important …. 3<br>Somewhat important (50%)…. 2	Q32 [ ]
33. **Do you think that too much salt or salty sauce in your diet could cause a health problem?**<br>Yes …………… 1<br>No …………… 2	Q33 [ ]
34. **Do you do any of the following on a regular basis to control your salt intake?**	
**34a. Limit consumption of processed foods**<br>Yes ……………. 1 No ……………… 2	Q34a [ ]
**34b. Look at the salt or sodium content on food labels**<br>Yes ……………. 1 No ……………… 2	Q34b [ ]

**34c. Buy low salt/sodium alternatives**<br>Yes .... [ ] 1 No .... [ ] 2	Q34c [ ]
**34d. Use spices other than salt when cooking**<br>Yes .... [ ] 1 No .... [ ] 2	Q34d [ ]
**34e. Avoid eating foods prepared outside of a home**<br>Yes .... [ ] 1 No .... [ ] 2	Q34e [ ]
**34f. Do other things specifically to control your salt intake**<br>Yes .... [ ] 1 Other: ____________ [ ] 9<br>No .... [ ] 2<br>*If Yes, go to 'Other'	Q34f [ ]<br>____________
The next questions ask about the oil or fat that is most often used for meal preparation in your household, and about meals that you eat outside a home.	
35. **What type of oil or fat is most often used for meal preparation in your household?**<br>Vegetable oil .... [ ] 1 Non in particular/mixture......... [ ] 5<br>Lard or suet .... [ ] 2 None used .... [ ] 6<br>Butter or ghee............ [ ] 3<br>Margarine.................. [ ] 4<br>Other: ____________ [ ] 9	Q35 [ ]<br>____________
36. **On average, how many meals per week do you eat that were not prepared at a home? By meal, I mean breakfast, lunch and dinner.**<br>_____meals per week	Q36 [ ]

37. **How often do you eat high-fat foods like fried chicken wings, curry puff, ngoh hiang, spring roll, samosa, Indian rojak, goreng pisang, yutiao, ham chi peng and roti prata, etc?**<br>Always (6-7 times per week) ……… ☐ 1<br>Often (2-5 times per week) ……….. ☐ 2<br>Sometimes (2-4 [1 time per week] times per month) … ☐ 3<br>Rarely (once per month)… ☐ 4<br>Never ………….. ☐ 5	Q37 [ ]
38. **How often do you eat processed meats like luncheon meats, bak-kwa, chinese sausage, smoked duck, spiced pork cube, minced pork with bean paste, etc?**<br>Always (6-7 times per week) ……… ☐ 1<br>Often (2-5 times per week) ……….. ☐ 2<br>Sometimes (2-4 [1 time per week] times per month) … ☐ 3<br>Rarely (once per month)… ☐ 4<br>Never ………….. ☐ 5	Q38 [ ]
39. **What type of dairy products do you usually eat/drink?**<br>Whole milk or full cream……. ☐ 1<br>Low fat………………………. ☐ 2<br>Non-fat or skim milk ………... ☐ 3<br>None …………….. ☐ 4	Q39 [ ]
40. **Do you remove skin from poultry and trim visible fat from meats?**<br>Yes ………….. ☐ 1<br>No ………….. ☐ 2	Q40 [ ]
41. **What type of bread or bread roll do you USUALLY eat?**<br>White bread (ordinary/enriched).. ☐ 1<br>Wholemeal bread ……… ☐ 2<br>A mixture of white and wholemeal bread … ☐ 3<br>Do not eat bread or bread roll at all… ☐ 4<br>Others : ________________ ☐ 9	Q41 [ ]<br>____________
42. **What type of rice do you USUALLY eat?**<br>White rice ……………... ☐ 1<br>Brown or red rice ……… ☐ 2<br>Do not eat rice at all …… ☐ 3<br>Mixture of white & brown or red rice… ☐ 4<br>Others : ________________ ☐ 9	Q42 [ ]<br>____________

43. **What type of sweetening agent do you USUALLY add to tea, coffee or other beverages?**<br>Sugar………………… ☐ 1 Do not add any sweetening agent at all… ☐ 3<br>Artificial sweeteners e.g. NutraSweet/ Equal/Saccharin-based syrups ……… ☐ 2 Others : _______________ ☐ 9	Q43 [ ]<br>____________
44. **How often do you drink sweetened drinks? (e.g. soft drinks, fruit drinks, packet drinks, cordials, yoghurt-based drinks and cultured milk drinks, etc.)**<br>_____ **days per week**	Q44 [ ]
45. **How often do you eat sweet desserts and snacks? (e.g. cakes, kuehs, jellies, candies, chocolates, cookies, ice-cream, etc.)**<br>_____ **days per week**	Q45 [ ]

SF-8TM Health Survey

This survey asks for your views about your health. This information will help you keep track of how you feel and how well you are able to do your usual activities. Answer every question by selecting the answer as indicated. If you are unsure about how to answer a question, please give the best answer you can. For each of the following questions, cross out a box that best describes your answer.	**Code**
46. **Overall, how would you rate your health during the past 4 weeks?**<br>Excellent …………….. ☐ 1 Fair ………..…….. ☐ 4<br>Very Good …………… ☐ 2 Poor ...…………….. ☐ 5<br>Good ……..………… ☐ 3 Very Poor …………... ☐ 6	Q46 [ ]
47. **During the past 4 weeks, how much did physical health problems limit your physical activities (such as walking or climbing stairs)?**<br>Not at all …………….. ☐ 1 Quite a lot …………… ☐ 4<br>Very little…..………… ☐ 2 Could not do physical activities … ☐ 5<br>Somewhat…………… ☐ 3	Q47 [ ]
48. **During the past 4 weeks, how much difficulty did you have doing your daily work, both at home and away from home, because of your physical health?**<br>Not at all …………….. ☐ 1 Quite a lot …………… ☐ 4<br>Very little…..………… ☐ 2 Could not do daily work …… ☐ 5<br>Somewhat…………… ☐ 3	Q48 [ ]

49. **How much bodily pain have you had during the past 4 weeks?**<br>None ………………….. [ ] 1<br>Very mild …………… [ ] 2<br>Mild ……………….. [ ] 3<br>Moderate …….……… [ ] 4<br>Severe ……………… [ ] 5<br>Very severe ………... [ ] 6	Q49 [ ]
50. **During the past 4 weeks, how much energy did you have?**<br>Very much ………….. [ ] 1<br>Quite a lot…………… [ ] 2<br>Some ………………… [ ] 3<br>A little …………… [ ] 4<br>None …………….… [ ] 5	Q50 [ ]
51. **During the past 4 weeks, how much did your physical health or emotional problems (such as feeling anxious, depressed or irritable) limit your usual social activities with family or friends?**<br>Not at all …………….. [ ] 1<br>Very little…………… [ ] 2<br>Somewhat……………. [ ] 3<br>Quite a lot …………… [ ] 4<br>Could not do social activities… [ ] 5	Q51 [ ]
52. **During the past 4 weeks, how much have you been bothered by emotional problems?**<br>Not at all ………….. [ ] 1<br>Slightly ……..……… [ ] 2<br>Moderately ……..……. [ ] 3<br>Quite a lot …………… [ ] 4<br>Extremely …………..… [ ] 5	Q52 [ ]
53. **During the past 4 weeks, how much did personal or emotional problems keep you from doing your usual work, school or other daily activities?**<br>Not at all …………….. [ ] 1<br>Very little…………… [ ] 2<br>Somewhat……………. [ ] 3<br>Quite a lot …………… [ ] 4<br>Could not do daily activities… [ ] 5	Q53 [ ]

Thank you for answering the questions. We really appreciate your time and assistance.
**Office Use only:**<br>**Interviewer Name_________**<br>**Measurements**<br>**Your Program Ambassador will conduct the following measurements:**<br><br>**Waist circumference** (cm) (record to the nearest 0.5cm) __________(cm)<br>**Hip circumference** (cm) (record to the nearest 0.5cm) __________(cm)<br>**Waist Hip Ratio** (record to the nearest 0.5cm) __________(cm)<br><br>**BMI [Weight/Height^2]_________**kg/m^2<br>Measurement: Wt _____kg<br>Ht______m<br><br>**Body fat percentage**<br>Measurement :_________ %

Program ambassadors: Please check through the questionnaire to make sure the participant has answered all of the questions.

Name of Participant: ________________________________

Home address: __________________________

Home phone no: ________________________ Mobile no: _________________________
