# Supplementary material for: Physical activity and nutrition intervention for Singaporean women aged 50 years and above: study protocol for a randomised controlled trial
Source: Trials. 2018 Apr 27;19:257. doi: 10.1186/s13063-018-2562-2 (PMC5923190; doi:10.1186/s13063-018-2562-2)
Supplement: Supplementary file 4 — Health survey: Mandarin. (PDF 553 kb) [file 13063_2018_2562_MOESM4_ESM.pdf]

办公室使用

健康调查

个人资料

代号

1. 您的岁数?

_______ 岁

Q1 [ ]

请在回答第 2 和第 3 题时只划一个格子

2. 您的种族?

华人…………………………………… ☐1　马来人………………………….. ☐2

印度人 …………………………… ☐3　其他______________________ ☐9

Q2 [ ] ____________

3. 下列哪个最能形容您所受过的最高教育?

小学或没受教育………………….. ☐1　高中或中学……………………. ☐2

专业文凭…………………………… ☐3　大学或高等教育以上………… ☐4

Q3 [ ]

4. 婚姻现状.

已婚……… ☐1　丧偶 ………… ☐2　单身 ………. ☐3　离婚…… ☐4

Q4 [ ]

5. 请问您有以下的任何吗? (划掉所有符合的)

心脏病…………. ☐1　中风 ………... ☐2　高血压…......................... ☐3

糖尿病……..…… ☐4　癌症 ……....... ☐5　高胆固醇…..……………. ☐6

骨质疏松症……... ☐7　关节炎 ……... ☐8　其它: _______________ ☐9

Q5 [ ] ____________

6. 您有服用以下任何的药物吗? (划掉所有符合的)

高血压药 (例如 Ramipril)……………………………………………………..……. ☐1

胆固醇药 (例如 Lipitor) …………………………………………………………… ☐2

血液凝结药/抗血小板药 (例如 Aspirin) ………………………………………………… ☐3

β 受体阻滞剂 (例如 Cartrol/Cartia) ………………………………………………… ☐4

其它: ____ ☐9

Q6 [ ] ____________

7. 您居住的住房类型。

2 房式政府组屋………………………………………………………………………… 1

3 房式政府组屋………………………………………………………………………… 2

4 房式政府组屋………………………………………………………………………… 3

5 房式政府组屋………………………………………………………………………… 4

其它: ____ 9

Q7 [ ]

体力活动

接下来我将会问您同常每周做各类体力活动所花费的时间。即使您认为自己不经常参加体力活动，请回答下列问题。<br>首先谈到工作中的体力活动。工作是指您必须完成的有酬或无酬工作，家务例如吸尘，抹地，搽地和逛街买杂货等。在回答以下的问题时，剧烈活动是指高负荷的体力活动并引起呼吸心跳显著增加。中等强度的活动是指一定负荷的适度体力活动并引起呼吸心跳轻度增加。	**代号**
**8. 您的工作需要做剧烈活动以致引起呼吸和心跳显著增加像是搬运拿或举重物，繁重的家务例如抹地，搽地板至少持续 10 分钟？**<br>是 …………………… 1<br>2 否 → ***跳到第 11 题***	Q8 [ ]
9. **您通常在一周有几天在工作中做激烈活动？**<br>_____ 每周天数	Q9 [ ]
**10. 您通常每天工作中做多长时间的激烈活动？**<br>_____ 每天几小时<br>_____ 每天几分钟	Q10 [ ]
**11. 您的工作需要做引起呼吸和心跳轻度增加的中等强度活动如快步走，提轻的杂货，扫地，清洗窗口至少持续 10 分钟？**<br>是 …………………… 1<br>2 否 → ***跳到第 14 题***	Q11 [ ]

12. **您通常每周有多少天工作时做中等强度的活动？**<br><br>_____ 每周几天	Q12 [ ]
13. **您通常每天工作时花多长时间中等强度运动?**<br><br>_____ 每天几小时<br><br>_____ 每天几分钟	Q13 [ ]
以下的问题不包括上述工作时的体力活动。<br>现在我想要问您平常的交通方式。例如，去上班、去购物、去市场、去宗教场所等。	
14. **您去某个地方时步行或骑脚踏车至少连续 10 分钟吗?**<br><br>是 …………………..... ☐ 1<br><br>☐ 2 否 → ***跳到第 17 题***	Q14 [ ]
**15. 您通常每周有多少天从一个地点到另一个地点步行或骑脚踏车至少持续 10 分钟？**<br><br>_____ 每周几天	Q15 [ ]
**16. 您通常每天在交通方面花多少时间步行或骑脚踏车(来回)?**<br><br>_____ 每天几小时<br><br>______ 每天几分钟	Q16 [ ]
以下问题不包括上述的工作和交通过程中的体力活动。<br>现在我想问您有关运动、健身和娱乐性体力活动（休闲）的问题。	
**17. 您进行引起您呼吸和心跳显著增加的剧烈的运动，健身和娱乐性体力活动（休闲）如跑步，游泳，跳舞如快步舞或萨尔萨，有氧运动，球类运动如足球，排球，擦网球或羽毛球至少持续十分钟?**<br><br>是 ……………………….. ☐ 1<br><br>☐ 2 否 → **跳到问题 20**	Q17 [ ]
**18. 您通常每周花多长时间进行剧烈的运动、健身或娱乐性（休闲）体力活动?**<br><br>_____ 每周几天	Q18 [ ]

**19. 您通常每天花多长时间进行剧烈的运动、健身和娱乐性体力活动？**<br><br>_____ 每天几小时<br><br>_____ 每天几分钟	Q19 [ ]
20. **您有进行引起您呼吸和心跳轻度增加的中等强度的运动、健身和娱乐性体力活动（休闲），如快步走、园艺、低有氧运动、用哑铃，用阻力带，气功或太极至少持续 10 分钟或以上吗？**<br><br>有 ………………….... ☐ 1<br><br>☐ 2 没有 → ***跳到问题 23***	Q20 [ ]
**21. 您通常每周有多少天进行中等强度的运动，健身和娱乐性（休闲）体力活动？**<br><br>_____ 每周几天	Q21 [ ]
**22. 您通常每天花多少时间进行中等强度的运动、健身和娱乐性（休闲）体力活动？**<br><br>_____ 每天几小时<br><br>_____ 每天几分钟	Q22 [ ]
以下问题是关于在工作时坐着、在家里在沙发上躺着、交通过程中、会朋友包括坐在桌前，与朋友一起坐着，乘坐轿车、巴士、地铁，阅读，打扑克或看电视，但不包括睡觉的时间。	
**23. 您通常每天有多少时间坐着或躺在沙发上？**<br><br>_____ 每天几小时<br><br>_____ 每天几分钟	Q23 [ ]

营养

以下问题是关于您平常会食用的水果与蔬菜。我这里有一张营养的海报，附有一些本地水果与蔬菜。一张图代表一个分量。当您回答这些问题时请想想去年的一周。	代号
**24. 您通常每周几天会吃水果？**<br>_____ 每周几天<br>若 0 天，请跳到问题 26。	Q24 [ ]
**25. 您通常在食用水果的那一天会食用多少分量？**<br>_____ 几个分量	Q25 [ ]
**26. 您通常每周有几天会吃蔬菜？**<br>_____ 每周几天<br>若 0 天，请跳到问题 28。	Q26 [ ]
**27. 您通常在食用蔬菜的那一天会食用多少分量？**<br>_____ 几个分量	Q27 [ ]
接下来的几题，我们想了解关于您饮食中的盐分。食用盐包括普通食用盐，粗盐例如海盐、咸汤料块和粉，和咸酱例如酱油或鱼露。<br>以下几题是关于您在用餐前的一刻所加的盐，关于在您的家是如何准备食物，关于吃含有高盐分的加工食物例如午餐肉、咸鱼、四川菜、腌萝卜、腌菜心、咸蛋、腊肠、腐乳和关于您控制您摄取的盐分。即使您认为您在食用低盐饮食，请回答以下的问题。	
**28. 您多常在用餐前的一刻或用餐时加盐或咸酱例如麻油？**<br>每次都会（6 到 7 天）………… ☐1　很少（一个月一次）... ☐4<br>常常 (2 到 5 天)… ☐2　从来没有…………….... ☐5<br>有时（一个月两到四次[一个星期一次]）……….. ☐3	Q28 [ ]

**29. 在您的家里煮或准备食物时多常加盐，咸调味料或咸酱？**<br>每次都会 （6 到 7 天）……..…. [1] 很少（一个月一次）… [4]<br>常常 (2 到 5 天) ……… [2] 从来没有……………... [5]<br>有时（一个月两到四次[一个星期一次]）………... [3]	Q29 [ ]
30. 您多常吃高盐分的加工食品？高盐分的加工食品是指已从自然状态所改变的食物例如包装咸小吃，罐装咸食包括泡菜、四川菜，在快餐店所准备的咸食和加工肉类等。<br>每次都会 （6 到 7 天）……..…. [1] 很少（一个月一次）... [4]<br>常常 (2 到 5 天)… [2] 从来没有……………... [5]<br>有时（一个月两到四次[一个星期一次]）………... [3]	Q30 [ ]
31. 您认为您食用多少盐或咸酱？<br>太多（天天）…………………. [1] 偶尔 (一个星期 1 到 3 次) ……. [3]<br>很多（一个星期 4 到 6 次）….. [2] 很少（一个月一次）……… [4]	Q31 [ ]
32. 对您来说降低您饮食中的盐分有多重要？<br>很重要………………. [1] 不重要…….……….…. [3]<br>有点重要(50%)……… [2]	Q32 [ ]
**33. 您认为若您的饮食中有太多盐或咸酱会造成健康问题吗？**<br>会……………….. [1]<br>不会…………….. [2]	Q33 [ ]
34. **您有定期做以下的任何来控制您所摄取的盐分吗？**	
**34a.** 限制食用加工食品<br>有……………… [1] 没有……………. [2]	Q34a [ ]

	办公室使用
**34b. 查看食品标签上的盐或钠含量**<br>有 …………….... 1　　没有 …………… 2	Q34b [ ]
**34c. 买低盐或钠的替代品**<br>有 ……………… 1　　没有 …………… 2	Q34c [ ]
**34d. 在烹饪时用除了盐之外的香料**<br>有 …………….... 1　　没有 …………… 2	Q34d [ ]
**34e. 避免吃在家以外所准备的食物**<br>有 …………….... 1　　没有 …………… 2	Q34e [ ]
**34f. 特地做别的事情来控制盐的摄取**<br>有……………… 1<br>没有 …………... 2<br>其它:_______________ 9<br>*若“有”，请到“其它”	Q34f [ ]<br>_______________
接下来的问题是关于在您家里准备食物时最常用的油和您在家以外所食用的饮食。	
**35. 在您的家庭里什么种类的油最常被使用来准备餐食?**<br>菜油……………… 1<br>腊或羊脂………….. 2<br>牛油或酥油………. 3<br>人造黄油………….. 4<br>没特别使用哪一种（参参使用）.... 5<br>没有使用……………………… 6<br>其它:____________________ 9	Q35 [ ]<br>_______________

36. 平均一个星期内您所食用的几餐不是在家里准备的？餐是指早餐，午餐和晚餐。<br><br>______一星期几餐	Q36 [ ]
37. **您多常吃高脂食物例如炸鸡翅、咖喱角、五香、春卷、印度罗惹、炸香蕉油条、咸煎饼和印度煎饼等**?<br>每次都会 （6 到 7 天）………. ☐1 很少（一个月一次）… ☐4<br>常常 (2 到 5 天)… ☐2 从来没有……………... ☐5<br>有时（一个月两到四次[一个星期一次]）………... ☐3	Q37 [ ]
38. **您多常吃加工食品例如午餐肉、肉干、腊肠、熏鸭、五香猪肉快、豆瓣酱猪肉碎等？**<br>每次都会 （6 到 7 天）………. ☐1 很少（一个月一次）… ☐4<br>常常 (2 到 5 天)… ☐2 从来没有……………... ☐5<br>有时（一个月两到四次[一个星期一次]）………... ☐3	Q38 [ ]
39. 您通常食用什么种类的乳制品？<br>全脂牛奶…………………… ☐1 无………………... ☐4<br>低脂牛奶……………………. ☐2<br>无脂或脱脂牛奶……………. ☐3	Q39 [ ]
40. 您有从家禽肉上去皮和修剪在肉上看得见的脂肪吗？<br>有……………….. ☐1<br>没有……………... ☐2	Q40 [ ]
41. 您通常食用哪种面包或面包卷?<br>白面包（普通/富）…..... ☐1 完全不吃面包或面包卷… ☐4<br>全麦面包 …………..……. ☐2 其它:________________ ☐9<br>白面包和全麦面包混合 …. ☐3	Q41 [ ]<br>____________

**42. 您通常使用哪种米饭？**<br>白米 ……………........... ☐1　　白米和糙或红米的混合… ☐4<br>糙或红米……………….… ☐2　　其它：________________ ☐9<br>完全不吃饭…………… ☐3	Q42 [ ]<br>____________
**43. 您通常在咖啡、茶或其它饮料中加什么种类的甜味剂？**<br>糖……………………….……….... ☐1　　完全不加任何甜味剂…… ☐3<br>人造甜味剂例如 NutraSweet/ Equal/Saccharin-based syrups……. ☐2　　其它：_______________ ☐9	Q43 [ ]<br>____________
**44. 您多常喝甜饮料？（例如汽水、果汁、包装饮料、甜果汁饮料、酸奶饮料和酸乳饮料等）**<br>_____ 一星期几天	Q44 [ ]
**45. 您多常食用甜品和小吃？（例如蛋糕、糕点、果冻、糖果、巧克力、曲奇饼、冰淇淋等）**<br>_____ 一星期几天	Q45 [ ]

SF-8TM 健康调查卷

这项调查会问您关于您对您的健康的看法。这项资料会帮您留意追踪您的感受和您多能做您的日常活动。请在回答每一题时选择所列的答案。若您无法确定如何回答其中的题目，请给您觉得最接近的答案。回答以下的题目时，划出最能形容您的答案的格子。	**Code**
46. **总的来说，您会怎么评估您过去 4 周的健康？**<br>非常好………………… ☐1　　还好…………………. ☐4<br>很好…………………… ☐2　　不好…………………. ☐5<br>好……………………... ☐3　　非常不好………….… ☐6	Q46 [ ]
**47. 在过去的 4 周，身体健康问题有多少限制您的体力活动（例如走路和爬楼梯）？**<br>完全不会…………….. ☐1　　非常多…………………… ☐4<br>很少…………………. ☐2　　完全无法做体力活动……. ☐5<br>有些…………………. ☐3	Q47 [ ]

**48. 在过去的 4 周，您在家里和家以外做日常工作时因健康问题面对多少困难？**<br>完全不会…………….. 1<br>很少………………….. 2<br>有些………………….. 3<br>很多……………………….. 4<br>完全不可以做日常工作….. 5	Q48 [ ]
49. **在过去的 4 周，您有多少的身体疼痛？**<br>没有…………………... 1<br>非常轻微……………… 2<br>轻微…………………... 3<br>中等………………… 4<br>严重………………… 5<br>非常严重…………… 6	Q49 [ ]
**50. 在过去的 4 周，您有多少精力？**<br>很多…………………… 1<br>蛮多…………………… 2<br>有些…………………… 3<br>一点………………… 4<br>没有………………… 5	Q50 [ ]
**51. 在过去的 4 周，您的身体健康问题或情感问题（例如觉得焦虑、抑郁或烦躁）是否限制您平时和家人或朋友的社交活动？**<br>一点也不会………… 1<br>很少………………… 2<br>有些………………… 3<br>蛮多…………………... 4<br>完全不能做社交活动… 5	Q51 [ ]
52. **在过去的 4 周，您有多少被情感问题困扰？**<br>一点也不会…………… 1<br>有点…………………… 2<br>还好…………………… 3<br>蛮多………………….. 4<br>非常多……………….. 5	Q52 [ ]
**53. 在过去的 4 周，有多少私人或情感问题阻扰您做平时的工作，学校或其它日常活动？**<br>一点也不会…………... 1<br>很少…………………… 2<br>有些…………………… 3<br>蛮多………………….. 4<br>完全无法做日常活动.. 5	Q53 [ ]

感谢您回答这些问题。我们由衷感谢您的时间与协助。

办公室使用而已:

面试官 ID _________　　程序员 ID __________________

参与者 ID _________

测量

您的活动大使会进行以下的测量:

腰围 (公分)
(测量至最接近的 0.5 公分)

__________(公分)

臀围 (公分)
(测量至最接近的 0.5 公分)

__________(公分)

腰臀比（公分）
(测量至最接近的 0.5 公分)

__________(公分)

身体质量指数 [体重/身高 ^{2}]_________公斤/米 ^{2}

测量: 体重 _____公斤
身高______米

身体脂肪百分比

测量:_________ %

活动大使：请检查问答卷以确保参与者已回答所有的问题。

参与者的名字：___________________________

家里电话：_______________________________

联络号码 (手提电话)：_______________________________

家庭地址:____
